# Injurious Effects of Underground Kitchens

**Published:** 1858-04

**Authors:** Frederick J. Brown

**Affiliations:** Chatham.


### INJURIOUS EFFECTS OF UNDERGROUND KITCHENS.

By FREDERICK J. BROWN, M.D., Chatham.

THE proper construction of dwelling houses is a matter of great importance to the public. The requisite conditions are salubrious site, dry foundations, efficient drainage, spacious apartments, and direct communication with the open air, so as to ensure thorough ventilation.

There is another condition, which is not generally noticed by writers ; it is this : that the basement story should be used only for cellar purposes, and not be divided into kitchens. The reasons are two-fold ; namely, (*a*) the darkness and damp to a certain degree inseparable from subterraneous apartments ; and (*b*) the exhaustion occasioned to those that occupy such kitchens by the frequent ascent and descent of stairs.

First, as to the effects of darkness and damp on the human body. Light is requisite to the colour, firmness, strength, and vigour of the body, and to the activity and cheerfulness of the mind. The more abundant the light, and the more direct that it is from the sun, the more decidedly are these good effects experienced. Light that is reflected has different properties from that which is direct. The actinic properties of sun-light are lost by reflection. Thus light that is reflected from a wall has but little value, compared with that which shines into a room direct from the broad face of heaven. The direct rays of the sun, then, are necessary to bodily and mental vigour. Those that live deprived of the sun's rays, feel a craving for stimulants such as is not experienced by those dwelling on open lands. Damp earth withdraws from the body its normal proportion of electricity, and thus occasions disorders that depend upon diminished nerve-force. These are ague, neuralgia, certain forms of rheumatism, epilepsy, chorea, and asthma, with some other affections, such as dyspepsia.

The servant-girls of London exemplify remarkably well these evil effects of damp; also the injurious results of deprivation of the solar rays. Their etiolated condition, and their breathlessness, show the anæmia (the impoverished state of blood) under which they suffer; and the functions peculiar to women are carried on imperfectly, or are absolutely suspended: hence the headache, the pains in the side, the palpitation, and the dropsical ankles, so frequently witnessed in this class. These circumstances are well known to the profession, and are especially familiar to those members who practise in the metropolis. Organic disease of the heart is originated by these causes in some instances.

The unhealthiness of underground tenements is shewn by the statistics of the city of Liverpool; the subterranean inhabitants being in a much worse sanitary condition than the supraterranean population.

Thus, then, the effects of darkness are disease, and not merely delicacy of structure; and the morbid condition is aggravated by the damp that is associated with the darkness.

(*b*). The next consideration is the exhaustion attendant upon the frequent ascent and descent of stairs. The necessity of answering the front door, and of ascending to the upper part of the house in the performance of domestic duties, obliges the servant to undergo much fatigue in running up and down stairs. The carrying of a burden up stairs is attended by a very much greater expenditure of strength than is the case in carrying it along a level. Girls that are breathless, owing to their anæmic condition, and oftentimes dropsical about the ankles, cannot afford the increased exertion that the circumstances necessitate: it is torture to them.

The value of land in London causes houses to rise vertically rather than to increase in dimensions horizontally. The same reason, namely, high value of land, is slowly but surely operating on the style of building in country towns. It will inevitably, if persisted in, bring about an approximation in the death-standard between the towns of small size and the great urban populations of this country.

It is the duty of physicians to point out the evil effects of subterranean apartments, and to impress upon the minds of builders, and upon the public generally, the injurious consequences of damp, dark kitchens, so placed that a multiplicity of stairs must be used in the performance of daily domestic duties.

In provincial towns, the wives of artisans and others of the working classes, occupying houses with basement kitchens, suffer much from exhaustion due to the necessity of frequently ascending the stairs. A woman gets up from her confinement pale and weak, from want of generous living, and perhaps, also, blanched by hæmorrhage: she is obliged to go up and down stairs some score of times in the day, and the consequence is a prolonged convalescence, with palpitation and other distressing symptoms, and sometimes even displacement of organs such as the womb.

Besides these ill effects upon women, the children are apt to meet with accidental falls down the stairs, and to harass their mothers by the watching necessary to prevent such accidents. Darkness and damp produce unfavourable effects upon growing children; and scrofula, and rickets, and consumption, and swelled bellies, are the result.

In the name, then, of humanity, let a stop be put to this sepulture of human beings, and to this treadmill exercise of women rendered weak and blanched by their premature interment.

---

